# Effectiveness of the Validation Method in Work Satisfaction and Motivation of Nursing Home Care Professionals: A Literature Review

**DOI:** 10.3390/ijerph18010201

**Published:** 2020-12-29

**Authors:** Iván Sánchez-Martínez, Raül Vilar, Javier Irujo, Duna Ulsamer, Dolors Cano, Celia Casaca Soares, Ángel Acevedo, Javier Jerez-Roig, Montserrat Celdrán

**Affiliations:** 1 Research Group on Methodology, Methods, Models and Health and Social Outcomes (M_3_O), Faculty of Health Sciences and Welfare, Centre for Health and Social Care Research (CESS), University of Vic-Central University of Catalonia (UVIC-UCC), 08500 Vic, Spain; ivan.sanchez@uvic.cat; 2 Fundació Privada Sant Andreu de Castellcir, Residència la Ginesta, 08183 Barcelona, Spain; 3 Validation Training Institute, Inc., P.O. Box 871, Pleasant Hill, OR 97455, USA; raulvil43@hotmail.com (R.V.); irujolizaur@gmail.com (J.I.); dunaulsamer79@gmail.com (D.U.); mdolorscano@hotmail.com (D.C.); 4 Centre for Interdisciplinary Applied Research in Health (CIIAS/IPS), School of Health, Polytechnic Institute of Setúbal, 2914-503 Setúbal, Portugal; celia.soares@ess.ips.pt; 5 Fundació Vella Terra, Residència, Centre de dia i Casal de Les Corts, 08029 Barcelona, Spain; angelacevedo.psico@gmail.com; 6 Research Group in Gerontology (GIG), Faculty of Psychology, University of Barcelona (UB), 08035 Barcelona, Spain; mceldran@ub.edu

**Keywords:** validation therapy, validation method, validation training, nursing, care, institutionalised, satisfaction, motivation, burnout

## Abstract

The purpose of this study was to carry out a literature review on the effectiveness of the validation method (VM) in job satisfaction and motivation of care professionals working with older people in nursing homes. The review was carried out in specialised databases: Scopus, PsychINFO, PubMed, Web of Science (WOS), Google Scholar, Scielo, and Cochrane Database of Systematic Reviews. 9046 results were obtained, out of which a total of 14 studies met the inclusion criteria: five quantitative, four qualitative, one single case series, two quasi-experimental and two mixed methods studies. The results of the analysed studies report that the VM can be an effective tool that facilitates communication and interaction in care, reducing levels of stress and job dissatisfaction among care professionals. The VM facilitates communication between professionals and older people with dementia, and improves the management of complex situations that may arise in care, directly influencing a reduction in work stress and increasing job satisfaction.

## 1. Introduction

The increase in life expectancy and the higher prevalence of neurocognitive disorders means there is a need to improve and innovate non-pharmacological strategies in order to ensure the quality of life and well-being of older people and their caregivers [1,2,3]. As a neurocognitive disorder progresses, one of the challenges is to be able to maintain effective channels of communication to identify the needs and emotions of older people with dementia and therefore be able to offer optimal, quality and humanising care [4,5,6,7].

Care for people with dementia can cause difficulties for care professionals, increasing their levels of stress, discomfort and job dissatisfaction [8,9,10,11]. Among the non-pharmacological strategies, the validation method (VM), created by Naomi Feil in the 1960s, is a specific communication method for older people with dementia [2,5]. Its main objective is to help professionals build a bridge to the internal reality of the disoriented person using the method’s verbal and non-verbal techniques, an empathic attitude and observation. Within this relationship, the emotions, lived experience, learnings, values, significant relationships, developed social roles, sensorial, physical, social and psychological losses of the person are all taken into account. The VM provides a theoretical framework that enables us to better understand the reasons behind the behaviour of the older person with dementia, promoting proper treatment focused on their real needs [12,13,14,15]. This relationship built on empathy and authentic listening helps the disoriented older people to express themselves and create closer, deeper and trusting relationship with the professional, which facilitates and improves communication between them, providing the older person with value, recognition and authenticity [15,16].

This methodology is linked to the need found in many residential centers to promote the development of professionals’ relational and socioemotional skills in order to improve positive and reciprocal relationships between them and the older people, which is a core aspect of care models like the well-known person-centred care model [17,18,19,20]. Furthermore, the VM has also shown benefits for professionals that are trained in and apply this method in terms of job satisfaction and motivation, as well as increasing their skills in relating and communicating with people with dementia or disoriented people [21,22,23]. In this way, being able to better understand the behaviour of disoriented people and equipping them with useful verbal and non-verbal communication tools for managing complex situations that arise in care could reduce the care workers’ stress levels [24,25,26,27,28], demotivation and job dissatisfaction [29,30]. However, scientific evidence has questioned its effectiveness and its theoretical principles–eminently humanistic–due to the difficulty in replicating experimental studies [31,32]. In a 2003 review on its effectiveness, significant methodological limitations were found in most of the studies carried out to date [32]. It is therefore necessary to update and review the available evidence to identify questions and areas under development for future studies on this type of intervention.

In this regard, this review is necessary given the increase in interest to improve the quality of care, giving priority to humanisation and the implementation of the person-centred care model [1,18,33,34]. This study therefore aims to carry out a literature review on the effectiveness of VM in the job satisfaction and motivation of nursing home care professionals.

## 2. Materials and Methods

A literature review was performed by peers of articles that included the effectiveness of VM in nursing home care professionals. The following databases were used for the review: Scopus, PsychINFO, PubMed, Web of Science (WOS), Google Scholar, Scielo and Cochrane Database of Systematic Reviews. The terms validation therapy, validation method, validation training, nursing, care, institutionalised, satisfaction, motivation and burnout were used. The search strategy used in the databases was: “(validation) and (therapy OR method OR training) AND (nursing or care or institutionalised) AND (satisfaction OR motivation OR burnout)”. In the Google Scholar database the following search was used: “(validation) and (therapy OR method OR training) AND (nursing or care or institutionalised) AND (satisfaction OR motivation) and (Feil)”.

The bibliographic references of the selected articles were analysed manually, in order to find other studies that could potentially be included for the review. In addition, documents from grey literature were included, as well as scientific articles, systematic reviews (SR), metanalyses and book chapters that met the inclusion criteria (Table 1).

The initial selection and subsequent screening were carried out by the principal researcher based on the title and results summary. Subsequently, the final selection was carried out by peers in a team six reviewers: five experts accredited by the Validation Training Institute in VM [35], and one expert in scientific publications in the field of relationships-communication between older people and professionals. The purpose of the study and their roles in the review were agreed upon with them in a separate meeting. A table was then constructed to include or exclude studies that could become part of the review, and their suitability was assessed. The reviewers focused on the type of intervention, individual or group, as well as its effect on professionals. Those studies that described the impact of VM on care professionals were included, as were articles that included residents as a target population but distinguished between the effect of VM on professionals and on residents. To establish consensus regarding the suitability of the articles, a third and, if there was any doubt with an article, a fourth reviewer was incorporated to decide whether to include or exclude it in the review.

For the evaluation of each article, the following were taken into consideration: the year and country of the study, the type of study design (experimental, quasi-experimental, qualitative, quantitative, mixed method, single case series, and literature review), outcome measures, intervention group (participants, age, gender and professional profile), type of intervention, evaluation of intervention and main effects on results in healthcare professionals.

## 3. Results

**Figure 1:**
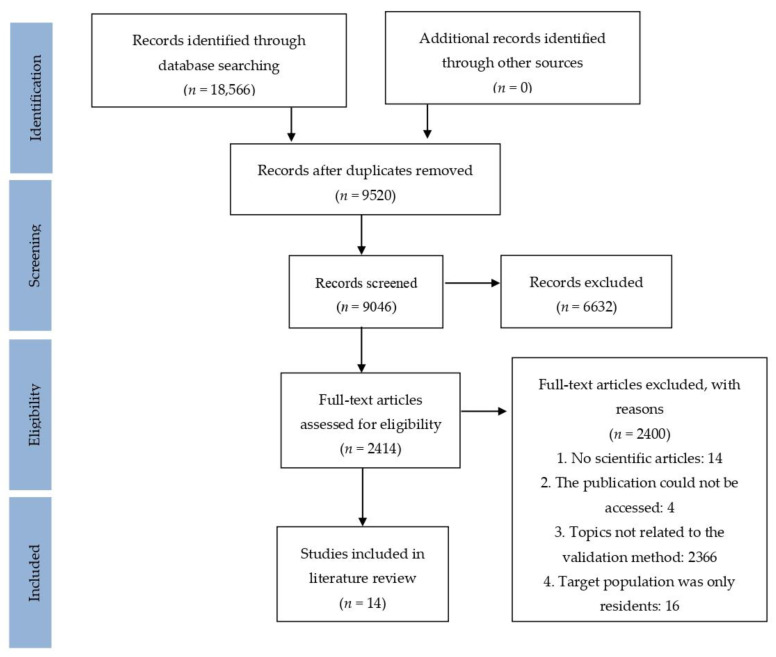
Literature analysis process.

Initially, 9046 results were obtained, out of which a total of 14 studies met the inclusion criteria (Figure 1).

With regards to the characteristics of older people that some studies included [5,22,23,24,25,27,28,36,37], they mostly coincided in age, gender and diagnosis of dementia (Table 2). Most of them included older people with a diagnosis of dementia, problematic care behaviours and mood problems. The average age varied between 83 and 90 years. Regarding gender, the study samples indicate a higher proportion of females that ranged from 67 to 100% [5,22,23,24,25,27,28,36,37]. Only one study [38] failed to report data on the composition of the sample by gender and age, but did do so on the diagnosis of dementia.

In contrast, the characteristics of the professional participants in age, gender, sample size and professional profile in the studies are heterogeneous (Table 2). Most of them included professionals with an average age ranging from 30 to 50 years. In terms of gender, the samples of the professionals in the studies have an over-representation of women that ranges from 66% to 100%. Only five (36%) of the included studies failed to provide information on the sample composition [5,28,36,37,38].

Regarding professional profile, 12 studies (86%) included nursing staff, made up mostly of nurses and auxiliary nurses, in the training of the VM and/or in its implementation [21,22,23,24,25,28,29,30,33,36,37,38]. Two studies (14%) also included, as well as nursing staff, management staff and other care professionals such as social educators, facilitators and psychologists [29,30]. Only one study (7%) included relatives as the target population of VM both in training and in its implementation [21]. Finally, Feil included in her study a trainer accredited in the VM by the Validation Training Institute (VTI).

These studies used different designs ranging from quantitative designs (5) [28,29,33,36,37], to qualitative (4) [22,23,25,30], mixed (2) [24,27], quasi-experimental (2) [21,38] and single case series designs (1) [5]. All the studies reported the effect of VM on the professionals, and one study explained its impact on a group of relatives [21] (Table 3 and Table 4). The country of origin of the selected studies was Italy [28,36], the United States [5,21,37,38], Sweden [22,23,24,25], the Netherlands [27], France [29,33] and Portugal [30].

The trial structure of the included studies that did training in the VM or analysed the effect on its implementation was, in two studies (14%), a single-arm trial where training in the VM was carried out [23,30]; in five studies (36%) a two-arm trial where training and implementation of the VM was done [22,24,25,29,33] and, in one study (7%), a three-arm-trial where the effect of training in the VM was analysed [38]. In contrast, in three studies (21%) a randomisation of the participants was performed for the effect of the implementation of the method in group sessions, as well as in sensory reminiscence [36], and one study (7%) was based on a VM-based emotion-oriented care training in comparison with a usual care training [27]. Similarly, Toseland et al. [37] conducted a study (7%) of three-arm trials with randomisation of the participants where VM training was carried out. Group sessions were performed and were compared with other strategies based on social contact care and usual care [36]. A study by Feil (7%) presented three case studies where the effect of the implementation of VM was analysed [5]. Finally, Canon’s study [21] (7%) trained professionals and relatives in VM, but there was no randomisation in the sample.

Although all the interventions were carried out in nursing homes, the duration and time of intervention varied. Most lasted between five and seven months [23,26,27,30], followed by a duration of between two and four months [28,36,38], and nine and thirteen months [22,24,25]. Only five studies lasted two to four days [21,29,30,33,37].

Regarding the variables of the professional studied, first, in the studies with quantitative measures, six studies (43%) quantified as outcome measures the effect of VM in three major areas [24,28,29,33,36,37]. First, two studies [29,33] evaluated stress reduction from the Maslach Burnout Inventory (MBI) [39] or the Neuropsychiatry Inventory (NPI) [40], used in studies 36 and 28, the work climate evaluated with the Creative Climate Questionnaire (CCQ) [41], used in study 24, or the Sheltered Care Environment Scale-Reality Version (SCES-R) [42], used in study 37, and how empathy influenced the prevention of burnout assessed by the Interpersonal Reactivity Index (IRI) [43] and the Jefferson Scale of Empathy (JSE) [44], used in study 33.

Second, in the studies with qualitative measures, five studies (36%) quantified as an outcome measure the satisfaction with the relationship and communication between the professional and the residents and between the latter and their families which was assessed through observations of the interactions between the professional, the residents and their families or with Hecht’s Interpersonal Communication Satisfaction Inventory (ICSI) [45], the Dementia Caregiver Quality of Relationship Inventory (DCQRI) [21] or Hudson’s CAM/CAF scale [46], used in studies [21,22,23,25,27,38]. The length of the conversations between the professional and resident, evaluated from the length of the interviews conducted pre-post training, management of problem behaviours, as well as work stress, were evaluated qualitatively through questionnaires prepared by the researchers themselves [21,22,23,25,30], or through the General Health Questionnaire (GHQ-28) [47], the Work Satisfaction Scale (W-BNS) [48], the Organizational Role Stress Scale (ORS) [49], the Neuropsychiatric Inventory (NPI) [40], the Cohen-Mansfield Agitation Inventory (CMAI) [50] or the Jalowiec Coping Scale [51], used in studies 27, 36 and 28.

Third, two quasi-experimental studies (14%) and one case series study (7%), quantified as an outcome result the effect of VM on satisfaction with communication between the professional and the residents and between the latter and their families, its use in the positive management of problem behaviours and increasing understanding of the behaviours of the disoriented person with dementia evaluated through Hecht’s Interpersonal Communication Satisfaction Inventory (ICSI) [45], the Dementia Caregiver Quality of Relationship Inventory (DCQRI) [21], Hudson’s CAM/CAF Scale [46], the Cohen-Mansfield Agitation Inventory (CMAI) [50] or semi-structured interviews [21,22,23,24,25,27,28,30,35,36,37].

Finally, despite the heterogeneity in the sample composition and in the structure and duration of the training and implementation, all the studies included in the review agree that training positively influences satisfaction, motivation, competence and self-efficacy of the care professional, improving their productivity and commitment to the organisation. Although there are only two randomised studies, the evidence suggests that training in and/or implementing VM can have a positive effect on healthcare professionals on a personal/professional, interpersonal and organisational level.

In the professional sphere there is a reduction in stress levels [22,26,27,28,29,30,33,36,37] and an increase in satisfaction, motivation, competence, confidence and happiness, contributing to a greater well-being [22,29,30,33].

Regarding the interpersonal sphere, there is an improvement in communication skills between the professionals and the disoriented person, which facilitate the relationship and care [21,22,23,25,26,27,30,38]. There is also an improvement in, and greater adjustment of, the response of the professional to the needs and behaviour of the disoriented person [5,21,25,38], promoting a positive management of behaviours that arise in daily care [22,24,37,38] and a reduction in the use of the therapeutic lying [30]. This produces an increase in the satisfaction of both the professional and the relatives in terms of communication and relationship with the disoriented person [21,23,25].

Finally, in the organisational sphere, there is a positive increase in the work climate [24,37] and also productivity and commitment to the organisation [29,33].

The purpose of this study was to analyse evidence on the efficacy of the VM on the job satisfaction and motivation of nursing home care professionals. Despite the limitations and methodological heterogeneity of the studies, most of the studies indicate that the VM provides benefits to care professionals, concluding that training and the correct use of the communication techniques that the VM provides are useful tools for relating and communicating with disoriented seniors, reducing burnout and negative work climate, and increasing their productivity and satisfaction, motivation and happiness at work [22,29,30,33]. This helps professionals to be more focused on the older person, on understanding the real needs that remain to be met and that give meaning to their behaviour [2,5,51]. In this regard, there is also an increase in professional competence as there is greater positive management of the problem behaviours that arise in care, the building of a closer deeper and more empathetic relationship with the disoriented person, given that knowledge of the meaning of their behaviour is increased, and care is based on the life history and personal needs of the resident [2,5,16,18,21,23,24,25,30,33,52].

In the studies analysed, the variables most analysed in the professionals are burnout, job satisfaction, competence in the positive management of problematic situations that arise in care, as well as improvement of the relationship and communication between the professional and resident. Implementing and/or training in VM may have positive work effects that can be related to job satisfaction and motivation. Therefore, the communication tools, relationship, and theoretical framework provided by the VM is highlighted in this type of intervention since it provides tools for developing relational competencies, facilitating the relationship and communication between the disoriented senior and the care professional. As a result, it is necessary to extend research on non-pharmaceutical strategies that provide care professionals with tools, as well as the use of VM with other pharmaceutical interventions, as is being done, for example, with the Emotion-Oriented Care Model [16,26,27,53].

The VM, which is in some ways congruent with the good practices that the person-centred care fosters, promotes a change in the culture of care where the abilities of the older person with dementia are valued and empowered, and where the professional moves from a role of being caregiver of a patient to being a caregiver of an expert in his/her own care [1,14,34,54,55]. In this regard, the aim of this article was to find out the effects of VM on job satisfaction and motivation, and the studies analysed suggest that training in or using VM may have positive effects on the well-being of professionals. The studies carried out on the effectiveness of VM have certain methodological limitations that the analysis of this review has shown. First, the lack of control both in terms of sample composition and follow-up and abandonment of some participants, the use of non-standardised instruments, the lack of sensitivity to behavioural changes, non-representative samples, no randomisation of the experimental groups and no control groups, as well as the difficulty in controlling the abandonment of participants. Second, some studies failed to clearly describe the structure of the training, and there were variations between the studies in the content and duration. Most were qualitative, quantitative and, to a lesser degree, mixed studies. It would be interesting to complement the quantitative data with the qualitative changes that can be observed in the professionals since they would provide more complete information about how training and implementing VM provides positive benefits for the care professional in his/her work. At the same time it is essential to carry out more robust studies on how VM provides effects on the professionals’ work, improving their professional competence, work climate, satisfaction, motivation, productivity and a reduction of absenteeism.

Finally, this review has a number of limitations: One is that there may exist a publication selection bias, since the initial screening of articles was not done by peers. Furthermore, the heterogeneity, lack of information on the sample composition or the type of intervention, samples under thirty participants in some studies, as well as methodological limitations, means that the results need to be taken with caution. In addition, there may be a general publication bias in the evidence analysed and a bias on the “negative” effects of the use of and/or training in VM. The authors of the review have found this publication bias difficult to control. With regard to its strengths, it should be noted that an extensive search on the VM was conducted, where any type of study that dealt with the effectiveness of VM on nursing home care professionals was included, both in terms of implementation of, and training in, the method.

## 4. Conclusions

Training in and/or implementing VM provides professionals with techniques of relationship and communication with disoriented older people that enables and helps them to manage their care, improving their quality of life and acting as tools that protect them against burnout, given that stress and overwork are reduced. In addition, it leads to greater empathy and provides professionals with a greater understanding of the meaning of the residents’ behaviour, promoting good treatment focused on the real needs of the older person with dementia (or disoriented).

More exploration of this method’s techniques is required, since it offers a field of communication and relationship that should not be underestimated and that, as the scientific evidence shows, generates positive effects on the satisfaction and motivation of care professionals. To do this, it is necessary to generate studies with standardised instruments, appropriate samples, and also include mixed methodology to complement the qualitative nuances that are generated in the relationship and communication between the professional and the person with dementia (or disoriented) with the quantitative data.

## Figures and Tables

**Table 1:** Study’s inclusion features.

Features	Inclusion Criteria
Font Type	Any type (articles, books chapters, theses, etc.)
End-Users	≥than 65 years with dementia in nursing homes
Professionals Language	Healthcare professionals Spanish, English, French
Intervention	Validation method (individual or in group)
Design Types	Experimental, quasi-experimental, qualitative, quantitative, mixed method, single case series, literature review
Time Period	Unlimited

**Table 2:** Description of demographic characteristics and professional profile.

Study		Target Population			n		Mean Age (Years) (SD)		Women (%)		Professional Profile
		P	R	F	P	R	P	R	P	R	
Deponte, Missan (2006) [36]		x	x		P: NR	R: 30	P: NR (NR)	R: 86.8 (NR)	P: NR	R: >80% *	Nursing Staff
Tondi et al., (2007) [28]		x	x		P: NR	R: 50	P: NR (NR)	R: 88.2 (NR)	P: NR	R: 82%	Nursing Staff
								GCr: 88.5 (NR)			
								GEr: 87.8 (NR)			
Toseland et al., (1997) [37]		x	x		P: NR	R: 88	P: NR (NR)	R: 88 (NR)	P: (NR) *	R: 75%	Licensed practical nurses, caregivers *
								GEr: 87.8 (6.0)			
								GEr: 87.3 (6.1)			
								GCr: 87.8 (7.6)			
Feil, (1995) [5]			x		P: 3	R: 3	P: NR (NR)	R: 89.5 (NR)	P: 66.6 %	R: 67%	Validation therapist
Finnema et al., (2005) [27]		x	x		P: 99	R:146	P: NR	R: 83 (NR)	P: 87%	R: 81%	Nursing aides, nurse, ward assistant, team leader
							GEp: 30.8 (8.0)	GEr: 83.8 (5.3)			
							GCp: 30.2 (7.4)	GEr: 83.6 (5.8)			
Söderlund et al., (2013a) [24]		x	x		P: 68	R: 11	P: 45.3 (NR)	R: 85.5 (NR)	P: 91.2% *	R: 81.8%	Registered nurses, licensed practical nurses, nurse aides
Söderlund et al., (2013b) [25]		x	x		P: 8	R: 11	P: 49.5 (NR)	R: 85.5 (NR)	P: 100%	R: 81.8%	Registered nurses, licensed practical nurses, nurse aides
Sördelund et al., (2011) [22]		x	x		P(A/B): 23	R: 29	P(A/B): 44.3 (NR)	R: 88 (NR)	P (A/B): 100%	R: 79.3%	Registered nurses, licensed practical nurses, nurse aides
Sördelund et al., (2016) [23]		x	x		P: 4	R: 4	P: 50.5 (NR)	R: 86.5 (NR)	P: 100%	R: 100%	Licensed practical nurses, nurse aides
Hergue et al., (2019) [29]		x			P: 29	NA	P: NR	NA			Caregivers, nurses, doctors, manager.
							GEp: 40.5 (9.8)		P: 80%		
							GEp: 40.2 (9.4)		P: 100% *		
Narme, (2018) [33]	Study 1 (S1)	x			P: 124	NA	P: 38.4 (11.3)	NA	P: 92.7%	NA	Caregivers and Nurses
	Study 2 (S2)				P: 122	NA	P: 39.2 (9.9)	NA	P: 95%	NA	
Fine, Rouse-Bane, (1995) [38]		x	x		P: NR	R:35	P: NR (NR)	P: NR (NR)	NR	NR	Nursing staff
Canon (1995) [21]		x		x	P/F: 58	NA	P/F: NR (NR)		P/F: 86%		Caregivers, resident’s family
Oliveira, Sousa (2020) [30]		x			P: 22	NA	P: 46 (11.9)		P: 95.5%		Direct care worker, care-home manager, administrative assistant, psychologist and animator

* the information was provided by the authors; P: Professional; R: Resident; F: Resident’s family; SD: Standard deviation; (A)/(B): They are studies that have two parallel samples in the same study or they carry out two different studies in parallel; GEr: resident experimental group; GCr: Resident control group; GEp: Professional experimental group; GCp: Professional control group; NR: not reported; NA: not applicable.

**Table 3:** Included studies on the effect of the implementation of VM in nursing home care professionals.

Study	Country	Methodology				Conclusions
		Design	Outcome Measures	Intervention Group	Intervention Type	
Deponte, Missan, (2006) [36]	Italy	Quantitative Design	MMSE, BANSS, NPI	Residents (r): RCT 1 GEr: VM implementation 1 GEr: SR group 1 GCr: no VM implementation Professionals (p): 1 GCp was formed by professionals	The GErs were conducted by two different facilitators, 2 days a week, at the same time. Each session lasted 45–60 min for 3 months. After 3 months, it was evaluated by the same battery test as the pre-treatment. For the GCp the intervention type was not specified.	(P): increased effect on the caregiver’s feelings, giving meaning to the residents behaviours.
Tondi et al., (2007) [28]	Italy	Quantitative Design	MMSE, BANSS, NPI	Residents (r): 1 GEr: VM implementation 1 GCr: no VM implementation Professionals (p): 1 GEp was formed by professionals	GEr carried out individual sessions in VM of 20 min, 3 times a week, and group sessions once a week of 50 min for 4 months. The GCr did not receive the VM intervention The GEp participated in the individual and group sessions. A follow-up was carried out for 1 week at the end of the intervention.	(P): reduction of stress levels in professionals.
Feil, (1995) [5]	United States of America	Single unique cases series design	MMSE	3 case studies	Weekly individual and group sessions	(P): The use of validation techniques helps professionals to grasp the reason for, and give value to, the emotions expressed by the residents.
Söderlund et al., (2013b) [25]	Sweden	Qualitative design	CPS	2-arm trial VM training programme	Individual sessions for 12 months, 2–3 times a week, at the beginning they lasted between 3–14 min and, at the end of the training, from 5 to 36 min.	(P): VM training of caregivers improves their response to the needs and behaviours expressed by residents. The programme contributes to improving the communication skills of caregivers with residents, increasing quality care.
Söderlund et al., (2016) [23]	Sweden	Qualitative design	CPS	1-arm trial VM training programme	Individual sessions 2–3 times a week for 5 months.	(P): Improvements in communication skills, increased conversations with residents from 3 min (at the beginning of the programme) to 36 min (at the end of the programme).
Hergue et al., (2019) [29]	France	Quantitative design	MBI, Karasek’s Scale	2-arm trial VM training programme	Burnout and social support questionnaires were sent to the heads of two residences. One group was formed in VM and the other group was not.	(P): The group formed in VM, the caregivers, feel more listened to, understood and supported; burnout is reduced and productivity, motivation, professional competence, satisfaction and commitment to the organisation are increased.

* the information was provided by the authors; P: Professional; R: Resident; F: Resident’s family; (A)/(B): They are studies that have two parallel samples in the same study or they carry out two different studies in parallel; GEr: resident experimental group; GCr: Resident control group; GEp: Professional experimental group; GCp: Professional control group; NR: not reported; **NA:** not applicable.

**Table 4:** Included studies on the effect of VM training on healthcare professionals.

Study	Country	Methodology					Conclusions
		Design	Outcome Measures	Intervention Group	Intervention Types	Evaluation	
Finnema et al., (2005) [27]	Netherlands	Mixed Design	(A) Quantitative GRGS, BIP, CSDD, CMAI, PGC, ASEP, BCRS, MMSE, GHQ-28, W-BNS, ORS, JCS Own questionnaire about facilities and illness (B) Qualitative QO	RCT Residents (r): 1 GEr: emotion-oriented care 1 GCr: usual care Professional (p): 1 GEp: emotion-oriented care training 1 GCp: usual care training	Training sessions in Model-Care Plan for 2.5 days for 7 months at GEp, combining the Model-Care Plan with the Emotion-oriented care based on VM. The GCp and GCr combined the Model-Care Plan with Usual Care	Follow-up and supervision 4 times for 1 day on empathic skills for the GE and usual care to GC. Pre-post training assessment, at 4 and 7 months.	(P): Reduction of stress levels and increased knowledge of tools that facilitate care. No differences in absenteeism and job competence were found in either group.
Toseland et al., (1997) [37]	United States of America	Quantitative Design	SPMSQ, VSI, MOSES SCES, CMAI, GIPB MDS	3-arm trial with RCT Residents (r): 1 GEr: VM implementation 1 GEr: SC group 1 GCr: no VM implementation, usual care (UC) Professionals (p): 1 GEp was formed by professionals	4-day training in the VM. 30 min group sessions of VM, SC, UC were held for 13 months, conducted by leading professionals from each group.	Weekly telephone follow-up and monthly physical supervision by a validation therapist. Pre-intervention assessment at 2 weeks after start of intervention and post intervention at 3 and 12 months.	(P): Professionals trained in VM have a positive increase in the management of problem behaviours at 3 and 12 months, understand the meaning of residents’ behaviours better, and have a more positive work environment.
Sördelund et al., (2011) [22]	Sweden	Qualitative Design	RAI, MDS, CPS	2-arm trial VM training programme	10 VM training sessions with supervision, spaced over one year, and practical VM training for at least 6 months between lesson blocks. 2–3 times a week.	Pre-post training assessment where participants received feedback from an accredited VM trainer throughout the year. Follow-up for 1 year.	(P): Improves the relationship with the resident, who improves the work environment. (P): VM is useful for handling difficult caregiving situations. Professionals were happier, less stressed and more confident.
Söderlund et al., (2013a) [24]	Sweden	Mixed Design	CCQ	2-arm trial VM training programme	Individual interviews after finishing the VM training programme after 12 months.	Analysis of the content of the interviews. Assessment of the work environment pre-post training.	(P): Increase in positive work climate scores. (P): The VM developed their communication skills and better handling of complex situations in care.
Fine, Rose-Bane, (1995) [38]	United States of America	Quasi-Experimental Design	QO	3-arm trial Residents (r): 2 GEr: VM implementation Professionals (p): 1 GEp was formed by professionals with VM training	The GEp carried out 6-h VM training sessions for 2 weeks.	Weekly follow-up sessions for workers for 3 months and pre-post intervention assessment.	(P): They found a 73% reduction in problem behaviours with the use of the appropriate technique for the disorientation phase and reduction of work stress.
Canon, (1995) [21]	United States of America	Quasi-Experimental Mixed Design	ICSI, DCQRI, CAM/CAF, VS	Non-RCT 1 GE: formed by caregivers and resident’s family. 1GC: formed by caregivers and resident’s family.	VM training sessions were held in 2 days of 7 h and pre-post training questionnaires were administered.	The pre-assessment was carried out before the training. The post-test assessment was carried out at the end of the training, up to 2 weeks after. There was no follow-up.	(P): increase in satisfaction with professional-resident communication after VM training. (F): the training gave them communication tools, a greater understanding of the expressions of their relatives and greater satisfaction in communication with their family member.
Oliveira, Sousa, (2020) [30]	Portugal	Qualitative Design	Own questionnaire about functioning session. Semi-structured interview. Focus Group.	1-arm trial VM training programme	4 group training sessions were held on the MV of 60–90 min for 6 months.	Content analysis on the effectiveness of training after each session and at 6 months through Focus Group. There was no follow-up.	(P): Increased empowerment, self-confidence and reduction in stress levels and the use of therapeutic lying.
Narme, (2018) [33]	France	Quantitative Design	MBI, IRI, JSE	S1: 2-arm trial 1 GE: formed by nurses. 1 GE: formed by caregivers.	Burnout, interpersonal reactivity and empathy questionnaire administered.	Correlation between the risk of burnout and the scores on the empathy scale and its comparison between nurses and aides.	(P) S1: The different cognitive and emotional aspects of empathy do not contribute in the same way to the appearance of professional burnout. There are no differences in empathy scores based on professional status.
			MBI, IRI	S2: 1-arm trial VM training programme	There were 3 days of MV training, 2 months of practical sessions, 2 days to discuss observed changes and 1 day to fill in post questionnaires.	Pre-evaluation before the start of the training. At 2 months there was a session to discuss changes observed in VM practice. After 6 months, the post questionnaires were administered. There was no follow-up.	(P) S2: Training brings benefits to workers, the quality of care and institutional functioning. Training contributes to burnout and stress prevention.

P = Professional; R = Resident; F= Resident’s family; O = Organisation; MMSE = Mini Mental State Examination; BANNS = Bedford Alzheimer Nursing Severity Scale; NPI = Neuropsychiatric Inventory; BIP = Behavioral Scale for intramural psychogeriatrics; GRGS = Geriatric resident goal scale; CSDD = Cornell Scale for depression; PGC = Philadelphia Geriatric Center Morale Scale; ASEP = Assessment scale for elderly patients; QO = Qualitative observations; BCRS = Brief Cognitive Rating Scale; GHQ-28 = General Health Questionnaire; W-BNS = Work Satisfaction Scale; ORS = Organizational Role Stress Scale; JCS = Jalowiec Coping Scale; CCQ = Creative Climate Questionnaire; CPS = Cognitive Performance Scale; RAI = Resident Assessment Instrument; MDS = Minimum Data Set; MBI = Maslach Burnout Inventory; IRI = Interpersonal reactivity index; JSE = Jefferson Scale of Empathy; ICSI = Hecht’s Interpersonal Communication Satisfaction Inventory; DCQRI = Dementia Caregiver Quality Relationship Inventory; CAF/CAM = Hudson CAM-CAF scale; VM = Validation Method; VS = Validation Skills; SPMSQ = Pfeiffer’s Short Portable Mental Status Questionnaire; VSI = Validation Screening Instrument; MOSES = Multi observational Scale for Elderly Subjects; SCES = Sheltered Care Environment Scale; CMAI = Cohen-Mansfield Agitation Inventory; GIPB = Geriatric Indices of Positive Behavior; MDS = Minimum Data Set.4. Discussion.
